# Sequential Cell-Processing System by Integrating Hydrodynamic Purification and Dielectrophoretic Trapping for Analyses of Suspended Cancer Cells

**DOI:** 10.3390/mi11010047

**Published:** 2019-12-30

**Authors:** Jongho Park, Takayuki Komori, Toru Uda, Keiichi Miyajima, Teruo Fujii, Soo Hyeon Kim

**Affiliations:** 1 Institute of Industrial Science, The University of Tokyo, 4-6-1 Komaba, Meguro-ku, Tokyo 153-8505, Japan; johopark@iis.u-tokyo.ac.jp (J.P.); tfujii@iis.u-tokyo.ac.jp (T.F.); 2 New Products Development Dept., Corporate New Business Development Office, NOK Corporation, 4-3-1 Tsujido-shinmachi, Fujisawa-shi, Kanagawa-ken 251-0042, Japan; komor@nok.co.jp (T.K.); udatoru1@nok.co.jp (T.U.); kmiya@nok.co.jp (K.M.); 3 Japan Science and Technology Agency PRESTO, Saitama 332-0012, Japan

**Keywords:** dielectrophoresis, deterministic lateral displacement, purification, single-cell trapping

## Abstract

Microfluidic devices employing dielectrophoresis (DEP) have been widely studied and applied in the manipulation and analysis of single cells. However, several pre-processing steps, such as the preparation of purified target samples and buffer exchanges, are necessary to utilize DEP forces for suspended cell samples. In this paper, a sequential cell-processing device, which is composed of pre-processing modules that employ deterministic lateral displacement (DLD) and a single-cell trapping device employing an electroactive microwell array (EMA), is proposed to perform the medium exchange followed by arraying single cells sequentially using DEP. Two original microfluidic devices were efficiently integrated by using the interconnecting substrate containing rubber gaskets that tightly connect the inlet and outlet of each device. Prostate cancer cells (PC3) suspended in phosphate-buffered saline buffer mixed with microbeads were separated and then resuspended into the DEP buffer in the integrated system. Thereafter, purified PC3 cells were trapped in a microwell array by using the positive DEP force. The achieved separation and trapping efficiencies exceeded 94% and 93%, respectively, when using the integrated processing system. This study demonstrates an integrated microfluidic device by processing suspended cell samples, without the requirement of complex preparation steps.

## 1. Introduction

Biochemical assays targeting cells suspended in body fluids are clinically used for diagnoses or prognoses. In particular, the analysis of cancer cells in blood samples is emerging as an important non-invasive medical method that is known as liquid biopsy. For instance, the assay on circulating tumor cells (CTCs), which are detached from the primary cancer tissue and circulate in the blood stream, has been widely investigated for the purpose of applying them as prognostic biomarkers in cancer research. However, unlike the conventional tissue-based biochemical assay, performing assays by using suspended cells involves important steps such as immobilizing the target cells on a substrate to facilitate biochemical processes, with the observation of target cells.

Recently, microfluidic approaches employing gravitational force, hydrodynamic force, or dielectrophoresis (DEP) have been developed to perform assays of suspended cells, at the single-cell level. In particular, various studies regarding the manipulation of mammalian cells have been performed using DEP. These studies involve the isolation and separation [1,2,3,4,5,6], sorting [7,8,9], and trapping [10,11,12] of cells inside microfluidic devices. We also developed a microfluidic device employing a microwell array to trap single rare cells [13,14]. Although a microfluidic device that employed DEP for cell trapping has been successfully demonstrated, there are several issues in the practical application of the systems for the analyses of various samples. First, the sample should be purified by prior separation such that only the targeted cells are trapped, using DEP. Second, it is always necessary to exchange the existing medium (e.g., culture medium) with the specific buffer to induce strong DEP movements. Currently, these processes are performed manually as pre-processing steps, including centrifugation and liquid handling, separate from the main DEP process. Thus, it is clear that pre-processing of samples should be integrated with the main analysis step to ensure an efficient analysis of the suspended cell samples.

Microfluidic devices have also been developed for the pre-processing of samples, which includes target cell isolation, exchange of medium, or concentration of samples. In particular, deterministic lateral displacement (DLD) has been extensively researched for the separation of micro particles in microfluidic environments. Ever since DLD was first reported for the separation of particles on the basis of size, in continuous flows [15], it has been widely used to separate various types of particles with several dimensions, such as micro/nano-sized particles [16,17,18,19,20], cells [21,22,23,24], DNA [15,25], and bacteria [26,27]. Those devices were mainly developed for sample preparation. Therefore, additional steps such as the collection of cells and the immobilization of suspended cells are still required for cell-based assays.

An effective strategy for the cell-based assay of the suspended cells in a single system is to integrate multiple microfluidic devices that have original functions, including pre-preparation of samples and downstream analysis. The integration of modulated microfluidic devices has been proposed previously and demonstrated by integrating the cell isolation module using acoustophoresis with the single cell array device [28,29]. However, a practical issue still exists for the integration of multiple devices of modules. The integrations in previous studies were achieved by directly bonding the outlet of one module to the inlet of another module. Although the integrated system has been demonstrated successfully, a more efficient and simpler method for the interconnection should be developed to ensure efficient integration. Moreover, the previously proposed system still requires pre-processing steps to exchange the medium to induce strong positive DEP, because medium/buffer exchange modules were not integrated into the system.

**Figure 1:**
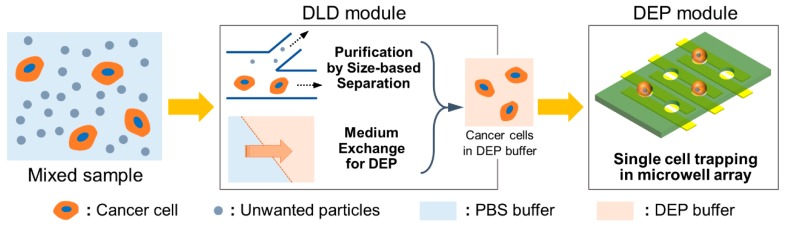
Schematic flow of processing samples via sequential cell-processing system.

In this paper, we propose an integrated system for sequential cell processing, using DLD arrays and dielectrophoretic trapping for the analyses of suspended cancer cells at the single-cell level. For the integration, two original modules were fabricated and efficiently integrated by connecting these modules through the interconnecting substrate that contained the rubber gaskets to maintain a tight interconnection between respective modules. Figure 1 depicts the schematic flow of processing samples in the sequential cell-processing system. To realize on-chip and sequential processing, we first introduced the sample into the DLD array module to exchange the cell medium with the DEP buffer. Simultaneously, the target cells were purified by the separation from the mixed sample via DLD array structures. The sample suspended in the DEP buffer continuously flowed into the DEP well array module through the interconnecting substrate. Subsequently, the cells were trapped in the microwell array at the single-cell level by inducing positive DEP with the electrodes patterned under the microwells. The separation efficiency of the prostate cancer cells (PC3) as well as the buffer exchange in the DLD module were evaluated. In addition, the trapping efficiency of the separated cells in the DEP module was also investigated. Finally, we confirmed the purification of PC3 cells from the mixed sample as well as the trapping of single cells by using the fabricated integrated device.

## 2. Working Principles

The DEP force (FDEP) acting on a cell can be expressed as:

(1) $$F_{DEP}=2\pi \varepsilon_{cell}a^{3}\mathrm{RE}\left(K\left(2\pi f\right)\right)\nabla {\left|E_{electric}\right|}^{2}$$

(2) $$K\left(2\pi f\right)=\frac{\varepsilon_{cell}^{*}-\varepsilon_{medium}^{*}}{\varepsilon_{cell}^{*}+2\varepsilon_{medium}^{*}}$$

where ε, a, f, and $E_{electric}$ are the permittivity, radius of the cell, frequency, and electric field strength, respectively. RE(K(2πf)) represents the real part of the polarization factor, also known as the real part of the Clausius–Mossotti (CM) factor. Here, $\varepsilon^{*}=\varepsilon +\frac{\sigma }{2\pi f}j$ indicates the complex permittivity, where σ is the conductivity; $j=\sqrt{\left(-1\right)}$, and the subscripts *cell* and *medium* represent the cell and the suspending medium, respectively. An important characteristic of DEP is that two different DEP phenomena occur according to the value of the real part of the CM factor. One is positive DEP whereby cells are attracted toward the strong electric field, when the value of RE(K(2πf)) is greater than 0. The other is the negative DEP whereby cells are repelled away from the strong electric field when the value of RE(K(2πf)) is less than 0. These two different movements were used to manipulate particles, including cells and microbeads [30,31,32,33,34].

In DLD, a specific arrangement of pillars is used to control the trajectory of particles based on their size, which causes the separation of particles that are larger or smaller than a specific diameter, which is known as the critical diameter. The critical diameter of DLD can be determined by using the rotation angle for pillar arrays and the size of lateral gaps between pillars, as follows:

(3) $$D_{C}=1.4g\varepsilon^{0.48}$$

where $D_{c}$ is the critical diameter, g is the lateral gap between pillars, and ε is the slope of a pillar array (ε=tanθ, where θ is the angle of gradient). In addition, the resolution of DLD separation depends on the lateral gap (g) and the row shift fraction (ε) of the pillar arrays. In this study, we focused on two characteristics of DLD, for the realization of a sequential cell-processing fluidic device. First, DLD is categorized as a passive separation technique that does not require an external field for separation processes. Therefore, it does not require a complex experimental setup for integration with other microfluidic systems. As a result, it is expected that the pre-separation of target cells for the DEP process can be realized at a minimum cost of design as well as fabrication, if DLD is integrated with the DEP device. Second, DLD is a microfluidic technique that is based on laminar flows. In laminar flows, the fluid flows in an orderly way and in parallel layers, which prevents mixing between adjacent layers. Hence, it is considered that on-chip sample preparation or processing on separated flows can be realized by controlling each flow stream, using DLD structures inside the microfluidic channels. For example, the staining of leukocytes via Rhodamine dye and a subsequent washing process was demonstrated by using DLD arrays [35].

## 3. Materials and Methods

### 3.1. Device Fabrication

**Figure 2:**
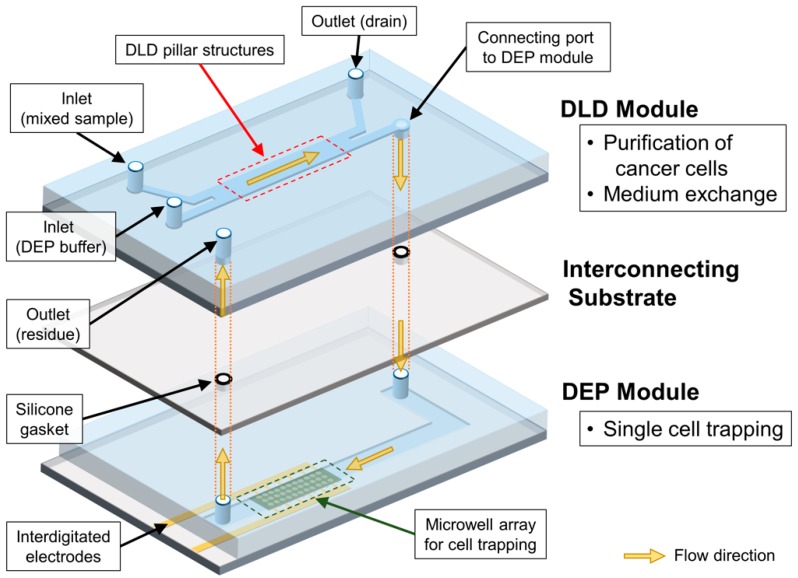
Schematic diagram of overall sequential cell-processing system.

The sequential cell-processing device mainly consists of two parts—a DLD module and a DEP module. We fabricated each module and subsequently integrated them by using the interconnecting substrate that has silicone rubber gaskets, as shown in Figure 2. Both the modules were fabricated using conventional photolithography. First, the DLD module was prepared using a polydimethylsiloxane (PDMS, Silpot 184, Dow Corning Toray, Co. Ltd., Tokyo, Japan) casting from the mold, which was fabricated using a negative photoresist (SU-8 3050, Kayaku Advanced Materials, Inc., Westborough, MA, USA). The PDMS channel with the DLD array was then bonded on a cleaned slide glass via oxygen plasma treatment, using a reactive ion-etching machine (RIE-10NR, Samco Co., Kyoto, Japan) In addition, the height and width of the PDMS channel in the DLD module were 60 μm and 2.5 mm, respectively.

Second, the DEP module was fabricated by patterning indium tin oxide (ITO) electrodes and SU-8 well arrays on a substrate. The shape of the interdigitated electrodes was initially designed using a CAD application. The patterns of the designed electrodes were then fabricated on a glass substrate, using conventional photolithography (Atsugi Micro Co., Ltd., Kanagawa, Japan). Thereafter, the microwell array was fabricated by using a negative photoresist (SU-8 3005, Kayaku Advanced Materials, Inc., Westborough, MA, USA) on the top of the patterned ITO electrodes. The diameter of the designed microwells was set as 24 μm, with a pitch of 100 μm. The total number of microwells in the array was 5554. The microfluidic channel for the DEP module was fabricated using polydimethylsiloxane (PDMS, Silpot 184, Dow Corning Toray, Co. Ltd., Tokyo, Japan), through a standard casting process. Here, the PDMS microchannel had a height of 60 μm, identical to the DLD module, and a width of 3600 μm. The substrate with the microwell array and the PDMS channel was then exposed to oxygen plasma and bonded together.

The interconnecting substrate was prepared using customized gaskets that were fabricated by screen printing a glass plate. Initially, holes were drilled in the glass plate, and these holes were used for connecting the DLD module on top, the glass plate in the middle, and the DEP module at the bottom. After the grooves for the gaskets were prepared at the top of the glass plate, the gaskets were then directly fabricated on the groove, using liquid-type silicone rubber (NOK Co., Tokyo, Japan). The thickness, inner diameter, and outer diameter of the printed gasket were 180 μm, 2 mm, and 4 mm, respectively. Finally, the surfaces of each layer were treated with oxygen plasma and bonded together, with the alignment of respective outlets and holes in the glass plate.

### 3.2. Sample Preparation

A human prostate cancer cell line, PC3 (obtained from the RIKEN Bio Resource Center, Ibaraki, Japan) was used for the model of a cancer cell in this study. The PC3 cells were cultured in a humidified incubator (37 °C, 5% of CO_2_ atmosphere). The culture medium was prepared using RPMI 1640 medium (Gibco™, Thermo Fisher Scientific Inc., Waltham, MA, USA), supplemented with fetal bovine serum (10%, Hyclone Laboratories Inc., Logan, UT, USA) and penicillin–streptomycin solution (1%, Gibco™, Thermo Fisher Scientific Inc., Waltham, MA, USA). The cultured cells were stained with CellTrace™ Calcein Red-Orange fluorescent dye (Thermo Fisher Scientific Inc., Waltham, MA, USA) and then harvested for the experiments. The concentration of cultured cells was adjusted to 5 × 10^5^/mL at harvest. Subsequently, a known number of cells were transferred to a mixed sample to obtain the right conditions for the DEP trapping, considering the number of microwells. In addition, two kinds of fluorescent polystyrene beads with a diameter of 0.5 μm (Fluoresbrite^®^ YG carboxylate microspheres and Fluoresbrite^®^ YO carboxylate microspheres, Polysciences, Inc., Warrington, PA, USA), the model for unwanted particles, were diluted with phosphate-buffered saline (PBS, Nacalai Tesque Inc., Kyoto, Japan) in a ratio of 1:100, resulting in a concentration of around 3 × 10^9^/mL, respectively.

The sample was prepared by mixing the prepared PC3 cells and the diluted polystyrene beads as described above. A DEP buffer with a low conductivity was prepared by mixing 10 mM of HEPES (Dojindo laboratories Co., Kumamoto, Japan), 0.1 mM of CaCl_2_ (FUJIFILM Wako Pure Chemical Co., Osaka, Japan), 59 mM of D-glucose (Sigma Aldrich Co., Ltd., St. Louis, MO, USA), and 236 mM of sucrose (FUJIFILM Wako Pure Chemical Co., Osaka, Japan). In addition, bovine serum albumin (BSA, 2% w/v, Sigma Aldrich Co., Ltd., St. Louis, MO, USA) was added to the prepared mixed sample and the DEP buffer solution to block non-specific cell adhesion. Thereafter, the prepared DEP buffer was sterilized via filtering, using a 0.22 μm membrane filter. All the buffers were freshly prepared for each experiment. The conductivity of each prepared buffer was measured using a portable conductivity meter (ES-71, Horiba, Ltd., Kyoto, Japan).

### 3.3. Experimental Setup

The integrated device was placed on the x–y translational stage of an inverted microscope (IX71, Olympus Co., Tokyo, Japan). The flow and samples were monitored using a digital CCD (charge-coupled device) camera (ORCA-R2, Hamamatsu Photonics K.K., Shizuoka, Japan) attached to the microscope. Three syringe pumps (MFS-SP1, Microfluidic System Works Inc., Tokyo, Japan) were used as the inlet for the DEP buffer, the outlet for the beads and culture medium, and the final outlet for the cell residue. To realize DEP trapping via positive DEP, a sinusoidal electric potential of 10 Vpp and 6 MHz was applied to the ITO electrodes, using a function generator (WF1948, NF Corp., Yokohama, Japan).

## 4. Results and Discussion

### 4.1. Deterministic Lateral Displacement (DLD) Module and Cell Purification

**Figure 3:**
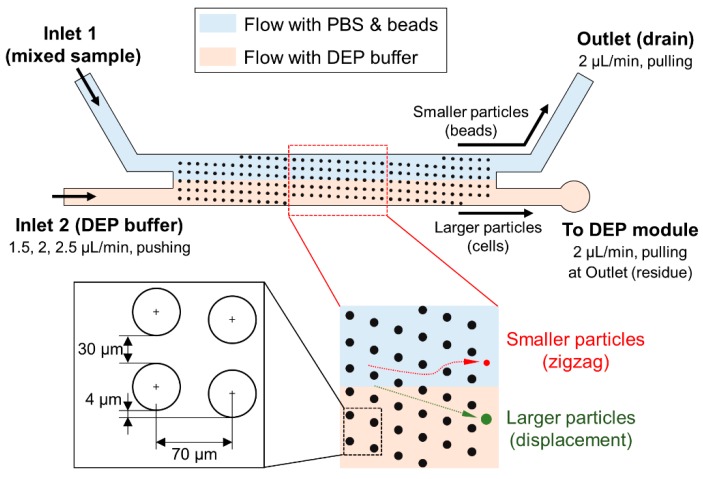
Details of the deterministic lateral displacement (DLD) module and details of pillar structures.

In this study, we designed the DLD module and pillar structures as shown in Figure 3. We set the critical diameter for the separation as 10 μm, assuming the purification of cancer cells from other type of particles. The diameter of the pillars, lateral gap between pillars, and distance between the centers of pillars were 4, 30, and 70 μm, respectively. As a result, the calculated value of critical diameter for our design was 10.63 μm. The average diameter of the cultured PC3 cells and polystyrene beads used in experiments were 22 μm and 0.5 μm, respectively. Thus, PC3 cells, which are larger than microbeads, are expected to move along the pillar structures in a displacement mode and finally flow continuously into the DEP module. On the contrary, the microbeads, which are smaller particles, are expected to move in a zigzag mode and flow into the drain of the outlet. In addition, two different streams—one with the PBS solution and another with the DEP buffer—flow separately through the DLD module because laminar flows are formed inside the microfluidic channel. As a result, the separated PC3 cells and the DEP buffer were finally joined downstream, when flowing into the DEP module.

First, we evaluated each function of the integrated device. Regarding the evaluation of cell purification in the DLD module, we measured the efficiency of PC3 cell separation from a mixed sample by changing the flow rate of Inlet 2 for the DEP buffer (Figure 3). We fixed the pulling flow rates of the outlet for the drain of the PBS buffer and beads, and the outlet for the residue collections of untrapped PC3 cells at 2 μL/min. The mixed sample was introduced directly into the hole of Inlet 1 through a customized reservoir. For the flow rate of Inlet 2, three flow rates; 1.5, 2, and 2.5 μL/min, were chosen for the evaluation of separation and trapping efficiencies. The separated PC3 cells were visually counted by using fluorescence at each outlet.

**Figure 4:**
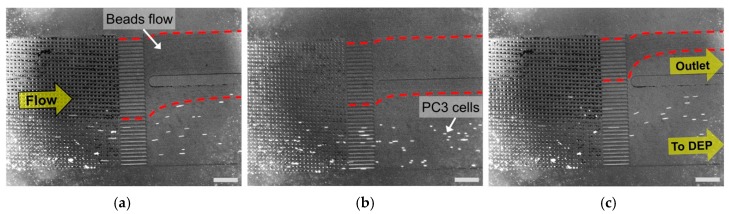
Microscopic images of cell purification in DLD module (scale bar: 500 μm). Prostate cancer cells (PC3): stained with CellTrace™ Calcein Red-Orange fluorescent dye (emission wavelength: 590 nm). Microbeads: Fluoresbrite^®^ YG carboxylate microspheres (emission wavelength: 486 nm). (**a**) Flow rate: 1.5 μL/min; (**b**) Flow rate: 2 μL/min; (**c**) Flow rate: 2.5 μL/min.

Figure 4 presents the microscope image near the outlet of the DLD module. PC3 cells, which has a different emission wavelength from microbeads, were confirmed by fluorescent microscopic images. The red dashed lines shows the trajectory and overall area of beads flow. It was evident that PC3 cells were purified from the mixed sample by the separation using DLD pillar structures. We observed that a few beads flowed into the connecting port to the DEP module, with a flow rate of 1.5 μL/min (Figure 4a). However, the beads flowing to the DEP module decreased gradually as the flow rate was increased to 2 and 2.5 μL/min. We calculated the separation efficiency of the PC3 cells by dividing the number of all separated PC3 cells with the number of total counted cells. Table 1 lists the results of the separation efficiencies with respect to three different flow rates. As shown in Table 1, it was quantitatively confirmed that the PC3 cells were successfully purified by the separation from the mixed sample in the overall flow rates. A separation efficiency exceeding 94% was achieved for all flow rates. It was also confirmed that the critical diameter, which was designed as 10.63 μm, corresponded well to the acquired separation results and efficiencies.

### 4.2. Verification of Buffer Exchange Using Dielectrophoresis (DEP) Module

In our study, we integrated the DLD module with the DEP module to achieve two purposes: the purification of target cells and buffer exchange. Here, buffer exchange means that the PBS constituent in a mixed sample is exchanged with the DEP buffer that was introduced from Inlet 2. In the DLD module, the flow including PBS is formed above the line connecting both branches because the laminar flow is generated as shown in Figure 3. Thus, the sample, except for the separated PC3 cells, finally flows into the outlet for drainage. In contrast, the DEP buffer from Inlet 2 flows below the PBS stream and proceeds into the DEP module with PC3 cells.

**Figure 5:**
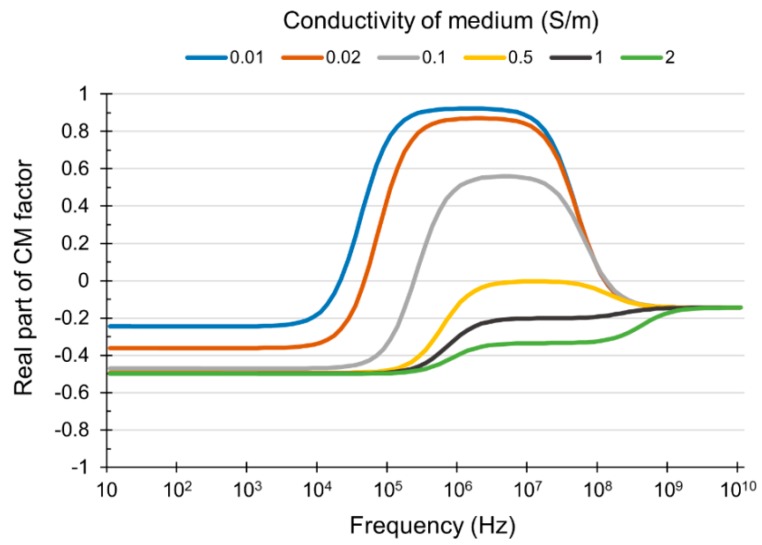
The relationship between the conductivity of the dielectrophoresis (DEP) medium and the real part of the Clausius–Mossotti (CM) factor.

Meanwhile, the PC3 cells are expected to be trapped in a microwell array by positive DEP in the DEP module. Considering a positive DEP, it is expected that the PC3 cells are not trapped in the microwell array of the DEP module without buffer exchange, because the high conductivity of the PBS solution causes a decrease in the magnitude of the real part of the CM factor, which results in the occurrence of a negative DEP (Figure 5).

Therefore, we verified the influence of the buffer composition for DEP trapping beforehand. For the verification, PC3 cells were introduced into a detached DEP module for trapping in a microwell array while changing the ratio between the PBS solution and the DEP buffer with a flow rate of 2 μL/min. An electric potential of 10 Vpp and 6 MHz was applied to the ITO electrodes. Table 2 shows the results of trapping efficiency with respect to different ratios of PBS and DEP buffer. It was observed that no trapping was achieved when the ratio of PBS was over 10%. Moreover, it was confirmed that even 2% of PBS can hinder the cell trapping and decrease the trapping efficiency compared with the result obtained using only DEP buffer. From the result, it was confirmed that the DEP trapping efficiency would be decreased unless the buffer is exchanged sufficiently at the DLD module.

### 4.3. Single-Cell Trapping by DEP

**Figure 6:**
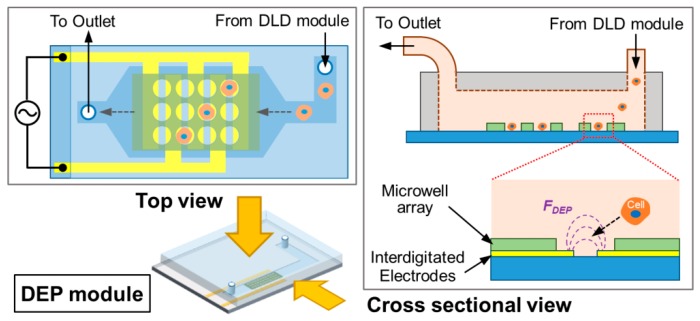
Schematic illustration of the DEP module using electroactive microwell array (EMA).

Finally, the PC3 cell trapping in the DEP module was evaluated by investigating the DEP trapping efficiency using the integrated device. The schematic details of the single-cell trapping device using electroactive microwell array (EMA) is shown in Figure 6. We used interdigitated electrodes aligned with through-hole structures in order to have both anodes and cathodes be exposed inside microwells [10]. The sample with only PC3 cells is flowed into the DEP module and applied to the DEP trapping by the positive DEP induced using interdigitated microelectrodes.

**Figure 7:**
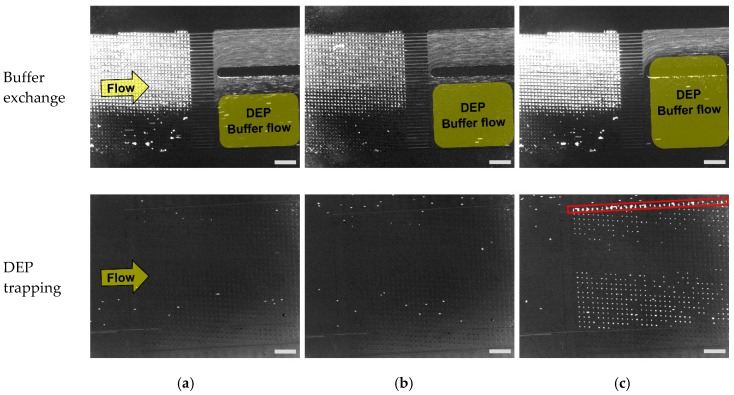
Buffer exchange in the DLD module (above), and the trapping of PC3 cells in a microwell array (below) for various Inlet 2 flow rates, scale bar: 500 μm. PC3 cells: stained with CellTrace™ Calcein Red-Orange fluorescent dye (emission wavelength: 590 nm). Microbeads: Fluoresbrite^®^ YO carboxylate microspheres (emission wavelength: 546 nm). (**a**) Flow rate: 1.5 μL/min; (**b**) Flow rate: 2 μL/min; (**c**) Flow rate: 2.5 μL/min.

In the previous section, we confirmed that the PC3 cells were purified from the microbeads by the DLD module. Here, we confirmed the buffer exchange at the outlet of the DLD module as well as the continuous cell trapping in the DEP module. All flows with beads and cells were observed or counted visually using fluorescence microscopy. Figure 7 shows the microscopic images of buffer exchanges and the cell trapping using DEP. To observe the buffer exchanges, we used different fluorescent beads having similar ranges of excitation/emission wavelengths of stained cells in order to visualize all the beads and cells simultaneously. As a result, the flow of beads or cells was confirmed as shown in Figure 7 (upper, Buffer exchange). It was observed that the flow with beads was clearly visualized by its flow shape due to the high concentration of beads (10^3^–10^4^ times bigger than that of cells). The yellow rectangles indicate the area where the DEP buffer flows, which shows that the flow of beads did not get into the DEP buffer stream.

As shown in Figure 7, the flow with beads including PBS is mixed with DEP buffer flow, and finally reaches the DEP module at the flow rates of 1.5 and 2 μL/min. When the flow rate was increased, the area of DEP flow grew larger which resulted in the decreased fraction of the beads flow with PBS. We also confirmed that all microbeads flowed into the outlet for the drain with the flow rate of 2.5 μL/min, which showed the removal of microbeads from the mixed sample. At the same time, it was confirmed that PC3 cells which were separated by DLD structures flowed into the DEP module as described in the previous section. From the results, we confirmed that the buffer exchange, as well as the purification of PC3 cells from a mixed sample, was successfully achieved by the DLD module.

Next, we confirmed that PC3 cells were trapped in a microwell array by DEP (Figure 7 (lower, DEP trapping)). Here, we observed that cells were successfully trapped in a microwell array at the flow rate of 2.5 μL/min. However, the cells were not trapped with the flow rates of 1.5 and 2 μL/min. From the results, it was verified that the buffer exchange was not sufficient for flow rates of 1.5 and 2 μL/min. In addition, the trapping efficiency was calculated by dividing the number of trapped cells in a micro well array by the total number of PC3 cells that flowed into the DEP module (Table 3). The result of trapping efficiency was well matched with the result of buffer exchanges because DEP trapping did not succeed with the two flow rates of 1.5 and 2 μL/min, which showed insufficient buffer exchange (Figure 7).

The results of DEP trapping and buffer exchanges, as well as the verification results of buffer composition showed that positive DEP did not occur with the buffer of increased conductivity that was caused by the inflow of PBS. On the other hand, we observed that several PC3 cells were trapped outside of microwell array structures (red rectangle, Figure 7c, DEP trapping)). It was confirmed that the electrodes of the designated part were exposed to the flow due to the misalignment of DEP electrodes and PDMS microchannel. As a result, cells were attracted to the exposed electrodes because the electric field of exposed electrodes was much stronger than that of the electrodes inside the confined area of a microwell array [13]. Thus, it was concluded that the alignment of electrodes and the channel structure should be performed accurately to avoid the loss of target cells. In conclusion, we confirmed that our developed sequential cell processing system can perform the separation of target cells with the buffer exchange in real time and finally trap the target cells continuously at a single-cell level with the flow rate of 2.5 μL/min.

## 5. Conclusions

In this study, we integrated the DLD and DEP module using the interconnecting substrate to realize a sequential cell-processing system. The purification of target cells and buffer exchanges were simultaneously realized and demonstrated using the DLD separation module. In addition, we confirmed the single-cell trapping of separated target cells in a microwell array using the DEP module and investigated the efficiencies of separation and trapping of target cells among three different flow rates. Finally, we confirmed that the purification with exchanging buffers and the single-cell trapping of target cells sequentially were achieved using our developed cell processing device. We believe that our study can minimize the pre-treatment steps in the sample preparation for single-cell analyses. Our device can be continuously used to handle or treat liquid bio-samples for liquid biopsy. For example, the purification by removing or separating red blood cells, platelets, and unwanted particles from blood samples, followed by the trapping of target cells, can be realized by using developed processing system. Moreover, we believe that our methodology to integrate two different types of microfluidic devices can be useful in the research fields of biochemistry and micro-nano systems.

## Figures and Tables

**Table 1:** Separation efficiencies of prostate cancer cells (PC3) cells in deterministic lateral displacement (DLD) module, at different flow rates.

Flow Rates (μL/min)	1st (%)	2nd (%)	3rd (%)	Average (%)	Standard Deviation
1.5	98.4	100	100	99.47	0.92
2.0	100	100	99.4	99.8	0.35
2.5	95.8	90.3	96.2	94.1	3.30

**Table 2:** Trapping efficiencies for different ratios of phosphate-buffered saline (PBS) and dielectrophoresis (DEP) buffer.

PBS:DEP (%)	1st (%)	2nd (%)	3rd (%)	Average (%)	Standard Deviation	Conductivity (mS/m)
PBS Only	0	0	0	0	0	1515.5
50:50	0	0	0	0	0	729.5
20:80	0	0	0	0	0	299.5
10:90	0	0	0	0	0	161.1
2:98	74.7	77	77.5	76.4	1.49	45.9
DEP buffer Only	96	89.8	86.6	90.8	4.78	16.51

**Table 3:** The trapping efficiencies in the DEP module.

Flow Rates (μL/min)	1st (%)	2nd (%)	3rd (%)	Average (%)	Standard Deviation
1.5	0	0	0	0	0
2.0	0	0	0	0	0
2.5	87.9	95.1	96.9	93.3	4.76
